# Integrating mind-body processes and motivational interviewing in health coaching: enhancing support for health behavior change

**DOI:** 10.3389/fpsyg.2025.1478525

**Published:** 2025-05-05

**Authors:** Ruth Q. Wolever, Rebecca Weinand

**Affiliations:** ^1^Osher Center for Integrative Health, Department of Physical Medicine and Rehabilitation, Vanderbilt University Medical Center, Nashville, TN, United States; ^2^Vanderbilt Health Coaching Program, Nashville, TN, United States; ^3^Weinand Coaching Services, LLC, Maryville, TN, United States

**Keywords:** health coaching, health and wellness coaching, motivational interviewing, mindfulness, guided visualization, Wheel of Health, Vanderbilt Health Coaching Funnel, IVA funnel

## Abstract

The global rise of chronic disease presents a need for effective prevention and treatment grounded in mind–body science and autonomy-promoting lifestyle interventions. Health and wellness coaching (HWC) has emerged as a new field as the evidence for it has grown. However, there continue to be significant discrepancies in how the HWC role is defined, trained, and practiced. HWC is an evidence-based approach integrating well-established behavior change theories and techniques to help individuals explore and sustain self-determined health targets. The National Board for Health and Wellness Coaching in the United States guides credentialling for the field and establishes minimum training standards and competencies for practicing health coaches. Foundational knowledge of the mind–body connection is newly included in these coach competencies. In this paper, we present the overall process of HWC used in the Vanderbilt Health Coaching Program, emphasizing how mind–body processes can be integrated with motivational interviewing. We specifically present three mind–body processes that we have entwined with motivational interviewing and iterated with over 700 trainees: use of mindfulness, the whole person Wheel of Health, and guided visualization. We also present two structural tools that overlay the mind–body processes and motivational interviewing: the Vanderbilt Health Coaching Funnel and it’s brief derivative for clinical encounters, the IVA (Importance Visioning Activation) Funnel. Each mind–body process and the two structural tools are described in detail as each promotes the underlying development of sustainable behavior change. Our aim is that these mind–body processes and structural tools will help clarify the evidence-based strategies upon which true coaching is developed and that other clinicians, researchers, and coaches will utilize them to empower their patients in pursuing their best health.

## Introduction

The study of mind–body medicine focuses on how the iterative relationships between the mind–body connection and behavior produce health and disease. Not only do biopsychosocial factors affect lifestyle, but lifestyle iteratively affects these factors creating patterns that lead toward or away from health and disease (Schulz et al., 2023). Lifestyle intervention is imperative to prevent and treat chronic disease including cardiovascular disease, diabetes, obesity, many cancers, and even anxiety and depression, in large part through impacting chronic inflammation (Bodai et al., 2018). As evidence for autonomy-promoting approaches to build healthy and sustainable behavior patterns has increased over the past decades, so have new job roles such as health and wellness coaching (HWC) (Wolever et al., 2016; Jordan et al., 2015). The growth of these roles and the global need to create and evaluate effective approaches for sustainable lifestyle behavior change establishes a unique opportunity to leverage mind–body processes (Schulz et al., 2023).

### Definitional problems

As with any rapidly-growing field, there continue to be significant discrepancies in how the HWC role is defined, trained and practiced. Since the term “health and wellness coach” is not protected by any title acts or regulations, even peer-reviewed papers evaluating “health coaching” often use these terms to describe interventions that do not use the foundational and empirically-based elements of HWC. Unfortunately, the problem is further confounded when apparently well-conducted systematic reviews of health coaching use as their inclusion criteria authors’ statements that the intervention was “coaching” (Sieczkowska et al., 2021; Rethorn and Pettitt, 2019) rather than clear indicators of well-established definitions for coaching (Sforzo et al., 2021). Most people—including trialists—do not understand the difference between coaching and other active interventions that are better referred to as clinical, educational or advising interventions (Wolever and Eisenberg, 2011). In fact, some interventions referred to as “health coaching” provide clinical assessment and recommendations, and are heavily educational in nature [e.g., (Karhula et al., 2015; Patja et al., 2012)]. HWC does not involve the process of diagnosis nor treatment. Further, HWC, by definition, provides only minimal education and does so in a specific autonomy-promoting manner (National Board of Medical Examiners and National Board for Health Wellness Coaching, 2022; National Board for Health and Wellness Coaching, 2022; Olsen, 2014). Instead, HWC uses well-established theories and evidence-based behavioral techniques to help individuals explore their self-determined health targets, elicit intrinsic motivation, and promote individual autonomy to identify and experiment with problem-solving techniques in an exploration for how to shift their lives in a way that only they can assess and sustain (Wolever et al., 2010; Wolever et al., 2011; Wolever et al., 2016; Matthews et al., 2024).

### Guiding the field

Despite equivocal findings (Sieczkowska et al., 2021; Rethorn and Pettitt, 2019) and challenges in the growing field of HWC, the HWC approach solidly rests on empirical work that has demonstrated effectiveness in helping individuals improve health behaviors (Matthews et al., 2024; Sforzo et al., 2020; Sforzo et al., 2018; An and Song, 2020; Boehmer et al., 2016; Kivelä et al., 2014; Ahmann et al., 2024; Radwan et al., 2019; Budzowski et al., 2019). To guide the field and ensure at least a minimal bar of HWC skill for practicing health coaches, the National Board for Health and Wellness Coaching (NBHWC) has emerged in the United States. Faculty from one of the NBHWC approved training programs, the Vanderbilt Health Coaching Program (VHCP), have been involved in collaborative efforts with other leading HWC programs/experts to bring clarity to what HWC is (and is not) (Wolever et al., 2016; Wolever and Eisenberg, 2011; Wolever et al., 2011; Sohl et al., 2021; Caldwell et al., 2013; Caldwell et al., 2020) and to strengthen the field through the development of evidence-informed tools and processes while also using them to train professionals in a reproducible intervention (Sohl et al., 2021; Caldwell et al., 2020; Wolever et al., 2017). As with any new and emerging field, rigorous research is needed to establish feasibility and effectiveness across different groups and settings; VHCP faculty is committed to contributing to the growing evidence demonstrating the potential of HWC (Wolever et al., 2010; Edelman et al., 2006; Wolever and Dreusicke, 2016; Wolever et al., 2022). In this paper, we present the overall process of HWC used in the VHCP, highlighting three mind–body processes and two structural tools developed and iterated with over 700 trainees in the VHCP and the Meharry Vanderbilt Health Coaching Program. It is our hope that these structural tools will help other researchers, clinicians and coaches recognize that the foundation of true coaching is MI, which can be leveraged with mind–body processes to further empower individuals in pursuing better health for themselves.

### Health and wellness coaching: models and background

Prior to presenting our overall HWC process and its integration with mind–body processes, we present the generally accepted methods of HWC with supporting background on their evidence base. We then highlight the integration of the mind–body processes that we use to enhance the behavioral change process. Specifically, we present how mindfulness training enhances coaching, a whole person model for self-assessment called the Wheel of Health, the use of guided visualization to assist with self-discovery, the Vanderbilt Health Coaching (VHC) Funnel as a motivational interviewing (MI) based tool for use in routine follow-up coaching sessions and its derivative, the Importance Visioning Activation (IVA) Funnel, for use in brief clinical encounters.

#### Health coaching models in general

While there are different health coaching models (Wolever et al., 2017; Smith et al., 2013; Purcell et al., 2021; Malecki et al., 2020), the evidence base is founded on the empirically derived definition of HWC on which the work of the NBHWC rests (Wolever et al., 2013). There is also consensus that HWC generally involves the following: an early “self-discovery” phase wherein the client explores their vision of improved health and well-being and sets a goal for the duration of the coaching (e.g., 6 months); a middle phase wherein the client explores a topic of their choosing and sets small action steps to take between sessions that move them toward their goal; and a final session where-in they review their progress and focus on maintaining improvement (National Board of Medical Examiners and National Board for Health Wellness Coaching, 2022; National Board for Health and Wellness Coaching, 2022). HWC rests on a handful of theories: Self-Perception Theory (Bem, 1967), Social Cognitive Learning Theory (Bandura, 1977; de la Fuente et al., 2023), Goal-Setting Theory (Locke and Latham, 2002), Self-Determination Theory (Ryan and Deci, 2000; Spence and Oades, 2011), and the Transtheoretical Model (Prochaska and Velicer, 1997; Prochaska and Prochaska, 2019). In addition, while many coaches may not realize it, HWC borrows heavily from the counseling style of MI (Miller and Rollnick, 2023). In fact, while additional evidence-based behavioral techniques are also used in coaching (Wolever et al., 2013; Simmons and Wolever, 2013), most models (unfortunately not all) utilize MI as a core component of health coaching (Budzowski et al., 2019; Linden et al., 2010; Butterworth et al., 2006).

#### Motivational interviewing as a foundational process

MI is “a particular way of talking with people about change and growth to strengthen their own motivation and commitment” (Miller and Rollnick, 2023). Effective application of MI requires an embodiment of the “spirit” of MI, dubbed the CAPE of coaching (CAPE: compassion, acceptance, partnership and empowerment) (Lanier et al., 2024). Effective application of MI also utilizes foundational communication skills, [i.e., open questions, affirmations, reflections, and summaries (OARS)], key MI processes (i.e., cultivating change talk and softening sustain talk), and an understanding of the four fundamental tasks of MI (Miller and Rollnick, 2023). Per the NBHWC competencies, health coaches must demonstrate competency, at a minimum, in the “spirit of MI” through creation of a patient-centered, empathic, non-judgmental, and empowering relationship in which the coach guides rather than dictates the change process (National Board of Medical Examiners and National Board for Health Wellness Coaching, 2022). They must also demonstrate competency in a number of communication skills that are also used in MI: deep listening and OARS (National Board of Medical Examiners and National Board for Health Wellness Coaching, 2022). Using these foundational communication skills and autonomy-supportive processes, coaches must be able to competently elicit change talk from clients and soften sustain talk (National Board for Health and Wellness Coaching, 2022). Across health coaching models and training programs, there is wide variability in the degree to which coaches are trained in MI, including how to cultivate change talk, soften sustain talk, and move clients through the four tasks of MI (Lundgren, 2024).

## Innovative foundation of the VHCP

The overarching VHCP model is unique in at least two ways. First, it walks individuals through the four tasks of MI over the course of the coaching relationship with all four tasks simultaneously being addressed in each ongoing session. While NBHWC provides approved training programs with a list of competencies in which entry level coaches must demonstrate proficiency, NBHWC does not specify a clear structure in which to utilize the skills or advise specifically on when coaches should utilize them in practice. Thus, many approved training programs teach MI as an underlying theory and introduce MI skills in isolation without a supportive structure. Coaches utilizing the VHCP session structure integrate the evidence-based communication skills and processes of MI in a systematic fashion. Upon completion of the program, health coaches can modify the structure to fit their varied settings; during training, the structure serves as an educational tool while they learn how various skills and processes work together.

The second way in which the VHCP model is innovative is that it entwines mind–body processes with MI in a manner that deepens client learning and ensures a whole person approach. Very little evidence or even description is available in the peer-reviewed literature regarding the overlay of mind–body processes and MI in coaching. There are multiple descriptions of health coaching from a whole person model, particularly in holistic nursing (Wolever et al., 2017; Purcell et al., 2021; Dossey et al., 2014; Bark, 2011; Delaney and Bark, 2019; Jordan, 2022). There are also a handful of studies that evaluate a coaching model that also uses mind–body techniques (Wolever et al., 2010; Edelman et al., 2006; Purcell et al., 2021; Gordon et al., 2023; Hudlicka, 2013; Malecki et al., 2020), but most do not even mention MI. In fact, literature searches in multiple databases [i.e., PubMed, PsycINFO, OVID (Medline and others)] using “mind–body” and “motivational interviewing” as key words reveal only minimal work that includes both mind–body processes and MI in intervention models. Furthermore, we could find no empirical work specifically evaluating the potential contribution of mind–body approaches to MI. In PubMed, we found four studies that assessed interventions that combined holistic breathing techniques with MI, and a case study using MI to address spirituality; none of these five involved coaching. Similarly, searching PsycINFO using the same key words revealed only 3 peer-reviewed pieces, all of which were irrelevant. Finally, a literature search using the database OVID (including Medline) and the key words “mind–body,” “motivational interviewing” and “coach” produced 33 links; of these, 14 were conference abstracts, 12 were non-empirical descriptions, 3 were reviews and only 4 were actual studies (or protocols) that included both mind–body approaches and MI; again, none empirically tested the contribution of mind–body processes. While HWC in the field is often delivered with the addition of mind–body processes, the use of mind–body processes to specifically augment MI has not received much empirical focus. Nonetheless, the recognition of the role of mind–body processes in behavior change is growing rapidly. This is evidenced by the NBHWC mandate that foundational knowledge of the mind–body connection is now needed by health and wellness coaches to meet the 2026 required competencies (National Board for Health and Wellness Coaching, 2024; see Table 1). The VHCP model is thus unique in that it integrates mind–body processes and whole person care with the evidence-based tasks of MI to promote sustainable behavior change.

**Table 1:** National Board for Health and Wellness Coaching required knowledge competency for mind-body connection (National Board for Health and Wellness Coaching, 2024).

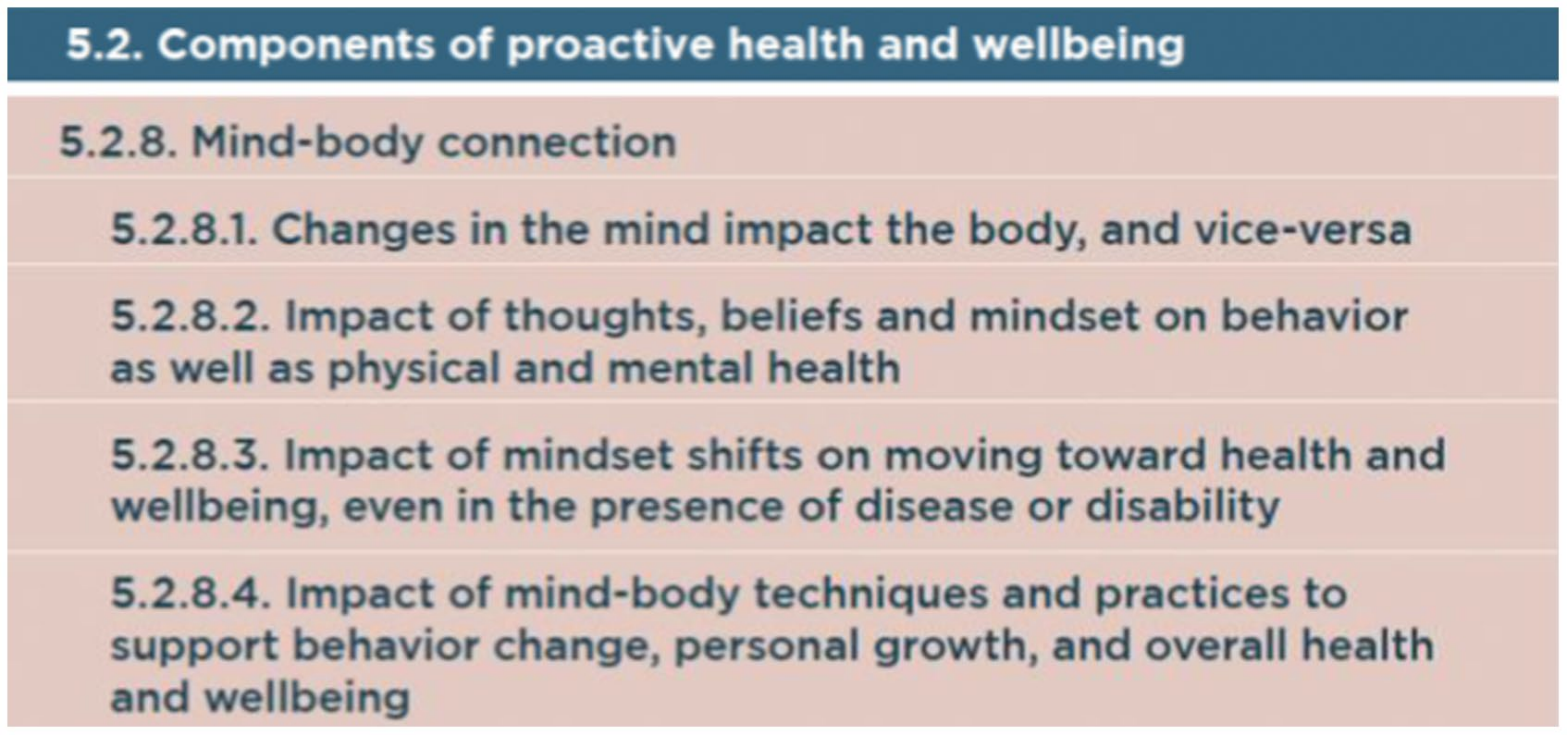

### Mind–body processes entwined with MI

The foundational change process taught in the VHCP is based on MI, with the integration of mind–body processes to enhance the client’s change process. First, mindfulness training is seminal for both the coach and to a lessor degree, the client. Second, a whole person integrative Wheel of Health is used to expand the client’s exploration in the self-discovery phase. Third, guided visualization is used to cultivate and amplify intrinsic motivation in the self-discovery phase as well as in ongoing sessions. In this visioning, the invitation to integrate sensory information is used to strengthen and deepen the learning to enhance confidence, self-efficacy and creative problem solving. In addition, multiple adult learning principles are woven throughout both the training program and the actual coaching process. For example, adults are self-directed learners whose wealth of experience facilitates learning (Cox, 2015). Learning should be relevant to their lives and is best delivered through hands-on involvement and practice (Cox, 2015). Two structural tools are also presented in this paper to ensure that core MI and mind–body processes are baked into the coaching practice: the VHC Funnel and the IVA funnel. The latter is a simplified version of the VHC funnel that can be used in 3–5 min during a clinical encounter.

#### Mindfulness training

Mindfulness is practiced by intentionally bringing close attention to the present moment with a gentle noticing, curiosity and non-judgment (Kabat-Zinn and Hanh, 2009; Siegel et al., 2009). Burgeoning evidence shows that practicing mindfulness has cognitive, emotional, and intrapersonal benefits, such as improving focus, increasing attention to percepts (thoughts, emotions, sensations) without elaboration, enhancing decision-making processes, and supporting cognitive flexibility (Didonna and Zinn, 2009). Mindfulness practice also promotes compassion, self-regulation, self-awareness (Gawande et al., 2019), presence (McCollum and Gehart, 2010), and interconnectedness, all of which are supportive of the coaching role (Rimban et al., 2024).

These same outcomes have made mindfulness an evidence-based strategy to support sustained behavior change as it promotes self-awareness, emotional regulation, self-regulation (Gawande et al., 2019), values clarification (Carmody et al., 2009), and thus, access to intrinsic motivation (Sohl et al., 2016). Self-awareness can be seen as a first step in developing the self-regulation required for sustainable behavior change (Brown et al., 2007), establishing the need for change and increasing insight into an individual’s own motivations and behaviors (Dossey et al., 2014). This insight, coupled with mindfulness, creates a space in which the individual is more likely to intentionally choose a behavior in alignment with their values system rather than out of habit (Roche et al., 2019; Schuman-Olivier et al., 2020). Furthermore, this lowers the cognitive effort around behavior change to nearly effortless (Roche et al., 2019; Schuman-Olivier et al., 2020; Redwine et al., 2022). In essence, mindfulness practice increases one’s awareness of how their relationships with their own thoughts, emotions and sensations bundle together to drive behavior (Wolever and Best, 2009).

The NBHWC includes mindful awareness as a foundational competency for coaches to promote therapeutic presence through active listening, “holding space,” empathy, and non-judgment (National Board of Medical Examiners and National Board for Health Wellness Coaching, 2022; National Board for Health and Wellness Coaching, 2022). While the depth of mindfulness training varies widely across NBHWC approved programs, qualitative and quantitative studies support its efficacy as a seminal part of the coaching process for both coach and client (Wolever and Best, 2009; Goble et al., 2017; Wolever et al., 2011; Spence et al., 2008). Hence, VHCP’s training centers on mindfulness as a necessary tool. Mindfulness is introduced as a means for coaches to personally deepen their ability to self-regulate, practice self-management, promote active listening, support therapeutic presence and develop positive rapport. Importantly, those with a mindful disposition are better able to deliver patient-centered care (Beach et al., 2013). VHCP trainees are first taught to use mindfulness to settle themselves, then later taught how to invite clients to be led in brief mindfulness practices at the beginning of sessions as a way to become fully present for the session. While always presented as a choice, practicing mindfulness with clients allows clients to experience being attuned with sensations and contextual cues, a practice they can use to support their own behavior change. Trainees are taught to use the MI tool “Ask-Offer-Ask” (Miller and Rollnick, 2023) to present the opportunity to be led in a mindful moment. The VHCP coaching process further promotes moment-to-moment awareness as coaches frequently inquire about client learnings, somatic experiences, and emotions throughout the coaching session.

#### VHCP model with mind–body processes used throughout the structure

In the VHCP, we utilize a semi-structured model designed to promote sustainable behavior change through the integration of mind–body processes, MI, positive psychology, and other evidence-based behavior change techniques. Aligning with NBHWC structure recommendations, the VHCP model provides a structure to help coaches learn to guide clients through the following: (1) early sessions in which they self-discover intrinsic motivation and set behavioral targets using goal-setting theories; (2) ongoing sessions where clients build momentum while experimenting with what works in their lives; and (3) a closing session which includes relapse prevention planning and celebrating the client’s progress and learning. The duration of coaching is highly variable and often determined by health plans or payors rather than science; it is often a 3–6 month timeframe.

##### The discovery phase

Also referred to by NBHWC as “early sessions” and by MI as the engaging task, the discovery phase occurs in the first one to two sessions. Early sessions are critical for establishing positive coach-client rapport and establishing the client-centered nature of health coaching (McCollum and Gehart, 2010; McKay et al., 2006; Krogh et al., 2019). Over the early sessions, a coach reviews the nature of the coaching relationship, explores the client’s motivation for seeking coaching, supports the client in a self-assessment, and explores the client’s optimal health vision, values and personal strengths. Unlike other healthcare relationships and the traditional MI model, VHCP early sessions typically last for 60 to 90 min, most of which is spent in self-discovery and the engaging task of MI.

###### Whole person Wheel of Health

**Figure 1:**
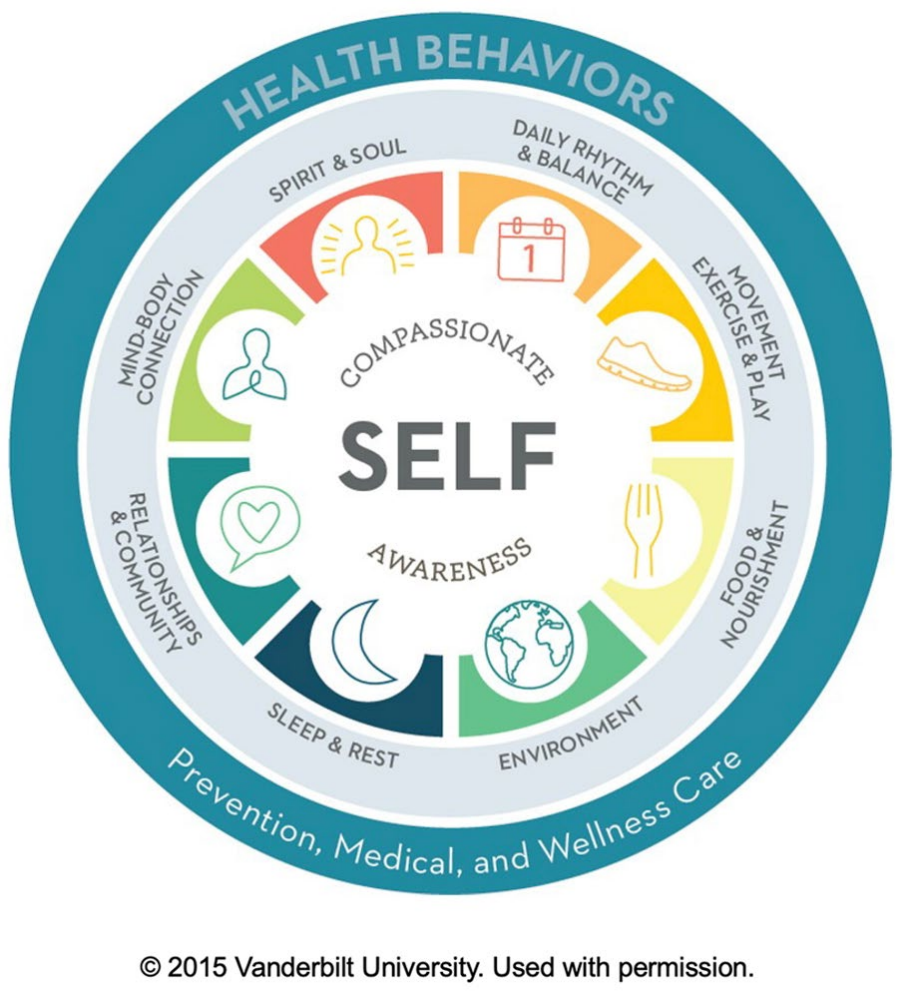
Wheel of Health (Courtesy of Osher Center for Integrative Health at Vanderbilt).

The VHCP model uses a whole person self-assessment (Wheel of Health) to support clients in identifying their current versus optimal state in nine different areas of health and well-being (see Figure 1). Clients rate their current satisfaction on a scale of 1 to 10, with 1 being “not at all satisfied” and 10 being “completely satisfied,” in each of the domains of health and well-being. In addition to indicating their current state in each domain, they indicate where they want to be in the same domain, and note their readiness for change in this arena. Coaching questions regarding the client’s desired state (where they want to be in a certain realm) begin to plant seeds of inspiration as clients imagine what would be best for them (Wolever and Dreusicke, 2016). Importantly, the use of this whole person self-assessment allows individuals to discover for themselves their own interrelationships between thoughts, emotions, body sensations and behavior. In essence, the self-assessment invites the client to think from a mind–body perspective about their daily routines and lifestyle habits. There are many different Wheels of Health, developed by different programs for different contexts; the one currently used in our program has been described elsewhere (Wolever et al., 2017).

###### Guided visualization

Following the exploration of the Wheel of Health, coaches using the VHCP model invite clients to a guided visualization exercise to imagine the experience of their default and optimal health visions. Again, the coach uses Ask-Offer-Ask to explain the process prior to leading the visualization. After the experience, the coach uses open questions and reflections to explore the client’s vision, values, and strengths, amplifying the gap between the client’s current and desired states. In doing so, the coach briefly moves to the evoking task of MI, cultivating preparatory change talk (DARN), building intrinsic motivation, and further nurturing the planted seeds of hope and possibility. In essence, guided visualization is used to magnify the gap between the client’s current behavior and their values as imagined in their desired state; this is a core process well-described in MI (Lanier et al., 2024). Creative guided visualization is a mind–body process with growing utilization in psychology, clinical care, integrative medicine, and behavior change therapies (Conroy and Hagger, 2018; Giacobbi et al., 2017). Guided visualization (also called guided imagery) has demonstrated effectiveness to promote healthy behavior change in eating (Conroy and Hagger, 2018), exercise (Cramer et al., 2014), substance use (Conroy and Hagger, 2018), and management of pain, anxiety, and stress (Giacobbi et al., 2017). The neural networks activated during guided imagery have the same effect on the body and mind as physically being present for an event or experience (Kosslyn et al., 2001). It is through these pathways that guided imagery impacts prevention and management of chronic disease. Thus, visualization can be used to increase an individual’s motivation, anticipated pleasure, anticipated reward, intention to change and likelihood of sustaining a behavior (Conroy and Hagger, 2018; Cramer et al., 2014).

###### End of the discovery phase: focusing, evoking, planning

To conclude the discovery phase, the client chooses an area of focus in which to work for the duration of coaching. The coach evokes the importance of this area using an importance ruler (also an MI tool) and elicits how the area connects to the client’s vision, personal values, and meaning (Goble et al., 2017; Wolever et al., 2011; Vorderstrasse et al., 2013). Early self-exploration is followed by goal-setting in which the coach guides the client to set an “umbrella goal” that covers the duration of the coaching relationship. Also known as a long-term goal, the self-selected umbrella goal defines a target behavior pattern the client wishes to achieve by the end of the coaching relationship. Per the VHCP model, NBHWC competencies, and MI strategies, the umbrella goal is as Specific, Measureable, Action-Oriented, Realistic and Timebound (SMART) (National Board of Medical Examiners and National Board for Health Wellness Coaching, 2022) as reasonable for the client’s knowledge and visioning at the beginning of coaching. Importantly, while many clients name outcome goals first, coaches support clients in translating their desires into behavioral goals or targets over which they have direct control (Bailey, 2019). A weight loss maintenance goal, for example, is turned into a specific physical activity goal. A “sleep better” goal is translated into sleep hygiene, and/or limited time in bed goals. A “feel less stressed” goal is translated into specific routines or behaviors the client commits to in order to lower their stress. Clients are also encouraged to have “approach” goals rather than “avoidance” goals (Oettingen and Gollwitzer, 2010; Bertholet et al., 2010). Instead of “limit beer intake at night,” the client might frame this as “after one beer, drink flavored water at night.” Or rather than “avoid screens one hour before bed,” the client might work toward, “reading, journaling, or practicing self-care one hour before bed.” According to neuroscience principles, focus on feared outcomes or avoidance tends to engage the amygdala (LeDoux, 2009; Frick et al., 2022). On the other hand, using approach goals that focus on building health, engenders creativity and iterative problem-solving, engaging the pre-frontal cortex and instilling a sense of imagination and hope (Cramer et al., 2014; de Souza et al., 2014).

In addition, the long-term goal(s) are tied to the client’s optimal health vision and values, increasing intrinsic motivation and likelihood of success (Locke and Latham, 2002; Kimsey-House et al., 2018). Goal-setting in HWC is distinct from that in MI in that goals are client-determined rather than shared, promoting client ownership, empowerment, and autonomy. Health coaches may bring in medical guidance from the client’s provider team, but do not push other-determined goals. They simply ask what the client thinks about their provider’s recommendations, and how, if at all, they want to integrate them. This goal-setting difference in coaching and MI can be significant, since in coaching, the promotion of client autonomy trumps medical advice in the immediate future. Anecdotal clinical reports suggest that clients tend to develop confidence from their success in making whatever changes they find highly relevant and for which they are ready (Ryan and Deci, 2000; Berkman, 2018). This confidence breeds success as one area of health and well-being tends to positively impact other areas. Because the VHCP model embraces whole person health, there are myriad places that the client may choose to focus. Hence, clients are encouraged to start with areas in which they feel most ready to work and even show excitement. After the specific long-term goal is set, coaches then support clients in setting successive SMART action steps (or short-term goals), moving to the planning task of MI and cultivating commitment and confidence from the client.

##### Middle phase: ongoing or follow-up sessions

In the middle phase of coaching (NBHWC routine, ongoing sessions), the VHCP model walks clients through the four tasks of MI in each session. Sessions are typically 30–45 min in duration and clients usually have 6 to 9 routine ongoing sessions, on average, depending on the clients’ needs, interest and long-term goals. VHCP coaches are trained to utilize the session structure as a guide to facilitate the coaching session. Coaches are equipped with mindfulness, communication skills, and MI strategies to effectively honor client needs, desires, and autonomy above adherence to a rigid structure. The structure of routine, ongoing sessions is outlined below and correlated with the four tasks of MI as shown:

###### Engaging

Assess client current state with an open question. Doing so helps the coach become attuned to the client and often opens the opportunity to practice mindfulness as a way to self-regulate and deepen awareness.
Invitation to a mindful moment, and leading of a brief practice if client desires.
Review of session agenda with input from client as desired.
Check-in on previous action steps with client permission

The coach uses open questions and reflections to elicit client successes and learnings with each action step, taking the opportunity to affirm client successes, insights, and efforts. Like much of HWC, this process draws on Self-Perception Theory in which clients hear their own narrative and begin to infer their qualities and skills from what they see themselves do and hear themselves say (Bem, 1967).
Learning is specifically elicited for each action step to support the client in developing an awareness of and self-efficacy in creating their unique behavior change journey, as explained by Bandura’s Social Learning Theory (Bandura, 1982).
If the client brings up barriers, challenges, or lack of action on an action step, the coach acknowledges the barrier to the extent necessary to establish and maintain rapport and understanding, but follows this with a reframe or inquiry regarding what the client learned that may be pulled forward in the behavior change journey. The check-in should occur at each session to build traction over time, allowing the client to link the action and learning from each experimental step as they move toward their umbrella goal. Consistently reinforcing and iterating action steps is important since behavioral repetition is one of the core components of establishing a new behavior pattern (Wood and Neal, 2016).
Learning is specifically elicited for each action step to support the client in developing an awareness of and self-efficacy in creating their unique behavior change journey, as explained by Bandura’s Social Learning Theory (Bandura, 1982).
As the check-in process is repeated frequently, clients also vicariously learn to look for success on which to build, and how to reframe failure as learning opportunities (Bandura, 1997).

###### Focusing

Coach elicits client-selected topic to discuss during the session. The topic may be related to an obstacle that arose in pursuing the client’s latest commitments to action steps. The topic may also be about what needs to happen next to continue building the desired behavior pattern. However, the topic may also be about something seemingly unrelated that has arisen and must be managed in order to keep attention and energy focused on the lifestyle goal, rather than habitual patterns of coping (Fournier et al., 2017; Schwabe and Wolf, 2009).

###### Evoking

Coach uses the VHC funnel, described in detail below, to evoke the client’s desire, ability, reasons, need, commitment, and activation for change.

###### Planning

Coach guides client to develop a SMART, behavioral action step that connects to client’s umbrella goal and/or optimal health vision. To set the client up for success, the coach uses open questions and reflections to investigate any needed environmental and interpersonal supports and a plan to engage each support. The coach invites the client to forecast potential barriers and supports them in establishing a contingency plan should those barriers arise (Bailey, 2019). Accountability and tracking plans are established, given the importance of self-monitoring in successful behavior change (Michie et al., 2009). Furthermore, tracking promotes client ownership of the new behavior and helps the client learn what does and does not work for them. Finally, the coach elicits the client’s confidence using a 1 to 10 scaling question and further explores how the client might increase their confidence if needed (i.e., < 7 on the 10-point scale).

###### Session closing

At the end of each follow-up session, the coach elicits client takeaways or key insights from the session. This process serves in the same manner as the check-in, emphasizing what the client is learning about their own behavior change process. The hope is that the client will not only clarify and attain their goals, but understand enough about the process that they can recontextualize the process for other changes.

##### Final session

The final session of the entire health coaching engagement follows a similar structure to that of the routine follow-up session, but in place of developing a next action step, the focus and the closing of the session are used to review what the client has achieved and what the client has learned about how they best change their own behavior relevant to their goal. In addition, the coach reviews the client’s maintenance plans asking the client to describe yellow flags that would indicate that the client may need more HWC or a stronger intervention to stay on track.

## VHCP structural tools

### Session focus: the VHC funnel

**Figure 2:**
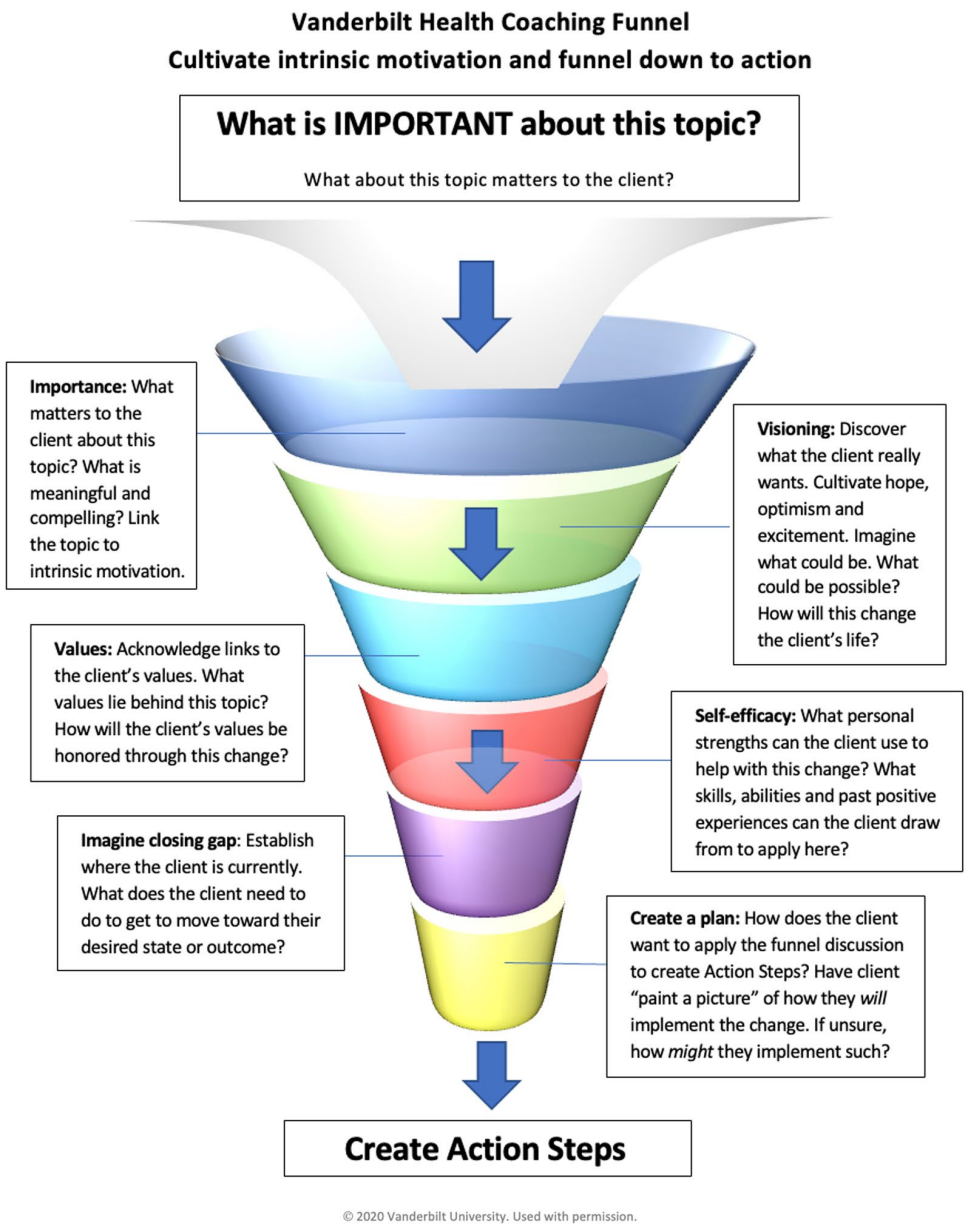
Vanderbilt Health Coaching Funnel (Courtesy of Vanderbilt Health Coaching Program).

As shown in Figure 2, a structural tool unique to the VHCP program is the VHC funnel, a framework utilized in the session focus to explore the client-selected topic and ensure that key MI tasks are covered with respect to the chosen topic while also allowing for the incorporation of mind–body processes. The VHC funnel intentionally integrates evidence-based strategies from MI to evoke preparatory change talk before moving to eliciting commitment change talk while simultaneously integrating visualization techniques and the use of multisensory information. By utilizing the VHC funnel, coaches explore the importance of change, connect desired change to a client’s vision and values (Caldwell et al., 2020; Wolever et al., 2017; Wolever et al., 2011), elicit client strengths that may serve them in the changes they desire, and invite client-determined steps. The coach uses powerful open questions, affirmations, reflections, and summaries throughout the funnel to cultivate change talk, soften sustain talk, and increase intrinsic motivation before planning for change with an action step.

#### Opening of the funnel

Coaching is a whole-person approach in which you start where the client is now and support them imagining into where they want to be, creating a space in which the client can move toward something rather than away from an undesirable behavior. The coach begins the session focus (at the top of the funnel) by eliciting what the client would like coaching around. The topic is always client-determined, rather than coach-selected, to maintain client autonomy.

#### Importance

Rather than moving directly into problem-solving, the funnel opens with an open question to elicit importance around the desired change. Questions may be straightforward, such as “What is important to you about this?” or “What matters to you about this area right now?” The intent is to elicit preparatory change talk and begin the exploration behind the “why” for change (Morris et al., 2022). Regardless of how specific the client-selected topic is, the coach explores how the area relates to their values rather than moving to problem-solving or goal-setting, as is often the case in typical healthcare settings. The coach listens for and reflects change talk, further exploring client desires and reasons for change with directional questions to evoke more change talk. Discussion of how the topic at hand connects to the client’s personal meaning, sense of purpose and values has important implications for health (Alimujiang et al., 2019; Mulahalilović et al., 2021; Friedman and Teas, 2023).

#### Visioning and values

Furthering this intrinsic motivation are two key components of implementing change: hope and confidence (Miller and Rollnick, 2023). While a client may cognitively understand reasons for change, change is unlikely without a belief in one’s own ability to change (Bandura, 1977) and a vision of what is possible (Conroy and Hagger, 2018; Cramer et al., 2014). By utilizing the VHC funnel, the coach invites the client to envision an optimal state in the selected area, using questions such as:

- How will your life be different when you have this just as you wish?
- What benefits will there be for you? For others you love?
- What will be possible for you when you make this change?
- If you could wave your magic wand, how would this area look in your life right now?
- When you envision yourself living as your best self in this area, what does that feel like in your body?
- What other areas in your life will be impacted when you make this change?

By describing their optimal state in detail, including the sensory component of it, the client is creating a felt sense of where their intrinsic motivation could take them (Patrick and Williams, 2012; Jack et al., 2013). Coaches ask clients to describe multisensory components of their vision, pulling for multisensory integration to enhance associative learning, anchoring in the potential impact of the desired behavior change (McGann, 2015; Lauzon et al., 2022). The coach may weave back and forth between linking the desired change to a client’s values and optimal vision, listening for and reflecting change talk from the client, amplifying client insights, and further exploring the desired change.

Throughout the session focus, the coach is listening for and reflecting stated and inferred values. Skillful coaches make explicit connections between the desired change and how it connects to client values, deepening desires, reasons, and need for change. The coach may explicitly ask the client how their values are connected to the desired change with an open question (“How does making this change support what matters most to you?”) or further explore stated or inferred values with meaningful reflections and powerful questions.

#### Building self-efficacy

A great deal of correlational research has shown individuals with higher degrees of self-efficacy are more likely to successfully implement and sustain behavior change (Bandura, 1982; Sheeran et al., 2016; Nezami et al., 2016; Kulik et al., 2019). More importantly, a meta-analysis of randomized controlled trials clarifies that it is the increase in self-efficacy that leads to improvements in both behavior intentions and behavior change. This meta-analysis included 50 studies (12,450 participants) on change in behavioral intention, and 90 studies (29,520 participants) on change in behavior itself, all of which included trials where participants were randomized to conditions where self-efficacy was empirically manipulated and tested for potential increases in intentions or behavior. Experimentally induced improvements in self-efficacy led to medium effect-sizes (Cohen’s *d* = 0.51) in intention to change and small to medium effect sizes for actual behavior change (*d* = 0.47) (Sheeran et al., 2016).

The VHC funnel moves deeper into cultivating preparatory change talk by inviting clients to verbalize their ability for change. Questions designed to promote self-efficacy and link client strengths to the desired change are built into the VHC funnel. Examples include:

- Which of your personal strengths can you use to help you with this change?
- How will your strengths help you move forward in this area?
- What have you done in the past to support success in a similar manner? How can you use that experience to help you move forward now?

Effective use of these strategies involves not only eliciting strengths and past successes from clients but making explicit links between a client’s strengths and successes and their ability to change moving forward. Throughout the funnel, the coach is listening for, reflecting, and amplifying client insight to promote empowerment and self-efficacy for change. Ideally, coaches will spend 12–18 min moving a client through the VHC funnel in a full-length coaching session. In using this tool with over 700 trainees, we have found that this amount of time consistently allows for cultivation of intrinsic motivation while also guiding clients toward clarity and readines to plan their next step(s) *(Commitment, Activation, Taking Steps)*.

#### Planning

As the VHC funnel narrows, the coach evokes mobilizing change talk by eliciting signs of their optimal vision and what next step is needed to move forward. Unlike shared goal-setting in traditional MI, the VHCP model promotes client-determined goals and action steps. The coach elicits a client-determined action step that feels realistic for the client to attempt in the next 1 to 2 weeks. The coach guides the client to set an action step that is SMART (Bailey, 2019), connected to their optimal health vision, and leads toward the long-term goal. To further move into planning, the coach invites exploration of what will help the client achieve the desired task, including potential environmental and interpersonal supports, potential barriers, contingency plans, accountability and tracking measures before assessing the client’s confidence using a 1 to 10 confidence scale. This planning process is completed for each action step to promote a sense of preparation, self-efficacy, and commitment to take the next step. These action steps aim to further concretize the commitment, activation and taking steps components of mobilizing change talk as described in MI.

Trainees have found that the VHC Funnel provides a structure for them to lean on as they develop their interpersonal and MI skills. There are many potential questions to be used in each section, but the intention and general process remain the same.

### IVA funnel for clinical encounters

**Figure 3:**
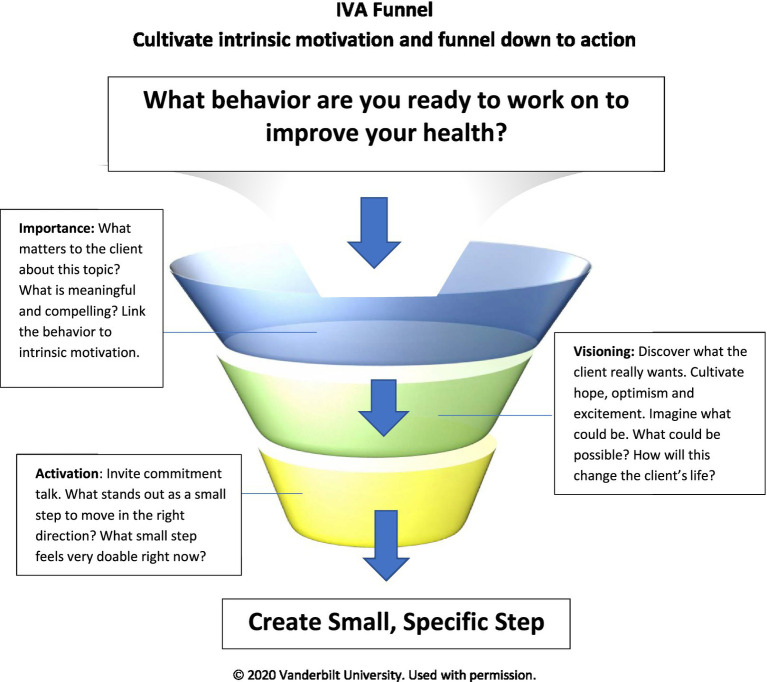
IVA Funnel (Courtesy of the Vanderbilt Health Coaching Program).

We recognize the need for these same evidence-based strategies to be integrated into brief clinical encounters to elicit patient motivation and activation for change. The current MI model of brief action planning (BAP) supports clinicians in developing well-supported SMART behavioral shared goals (Cole et al., 2023). However, the BAP does not address importance, values, visioning, or fully patient-centered goals. To this end, we have developed the IVA (Importance, Visioning, Activation) funnel as a structural tool for clinicians to explore personal importance of the change to the patient, elicit their vision, and support their activation in a brief clinical encounter while maintaining a patient-centered approach (see Figure 3). To begin, the clinician elicits from the patient a health behavior they are ready to change. Importance is then assessed using open questions and reflections, linking the desired change to intrinsic motivation, the patient’s desires, needs, or reasons for change. Visioning questions follow to invite the patient to consider how the desired change will benefit their life. The intent in visioning is to cultivate hope and optimism around the desired change. Finally, action-oriented questions activate the patient toward change, inviting commitment before supporting the patient to set one small, specific action step. Clinicians can use the IVA funnel in less than 5 min while still cultivating intrinsic motivation, hope, optimism, and commitment for change. Unlike BAP and other action planning models, the IVA funnel is largely patient-directed and emphasizes strategies to deepen motivation and commitment for change over specific action planning.

## Limitations and future directions

The integration of mind–body processes in coaching is happening across the HWC field. Nonetheless, it has not been well-described in the peer-reviewed literature, particularly in terms of its integration with MI. Further, the integration of mind–body processes and MI has not been studied empirically in coaching. The contribution of these processes to specifically augment MI, as well as health coaching in general, needs significant further investigation. While our trainees report that the tools are useful with diverse patient populations in varied clinical settings, neither has been systematically studied. It is our hope that the rationale and justification explained in this paper will support moving these important studies forward.

It is important to note that our integration of mind–body processes with MI and other behavioral change techniques aimed to support coaches who practice in healthcare settings in which patients have sought out or specifically been referred for health coaching (versus a clinical encounter). Except for the IVA funnel, these processes need further iteration to be used in clinical settings where time is quite limited. Even in the coaching setting, the Wheel of Health exploration can take multiple sessions when used with patients with complex health needs who are very incapacitated. The beauty of the Wheel of Health, however, is that it usually works with even those with low intrinsic motivation for behavior change. With the broad exploration of one’s life that is inherent in use of the Wheel of Health, almost everyone finds some area they wish to change.

## Conclusion

Knowledge of the mind–body connection is becoming more important in HWC. Mind–body processes are easily integrated with MI and other evidence-based tools to support behavior change in both HWC training and in providing coaching. Mindfulness supports the learning process for the coach and the behavior change process for the client. Client self-discovery using a whole person Wheel of Health provides the opportunity for the client to consider a broader context as they choose among multiple life domains and experiment with behavior change processes that will be sustainable in the context of their full lives. Guided visualization deepens the cultivation of intrinsic motivation and allows clients to link their optimal visions to multisensory learning. This intrinsic motivation is further supported by self-efficacy strategies to help clients implement and sustain behavior change. Finally, the VHC funnel provides a clear framework that ensures core MI processes are used in coaching, and integrated with the mind–body processes discussed. A simplified version (the IVA funnel) can be used in a clinical context when time is tight.

## Data Availability

The original contributions presented in the study are included in the article, further inquiries can be directed to the corresponding author.
